# Microbial Nanotechnology: Challenges and Prospects for Green Biocatalytic Synthesis of Nanoscale Materials for Sensoristic and Biomedical Applications

**DOI:** 10.3390/nano10010011

**Published:** 2019-12-18

**Authors:** Gerardo Grasso, Daniela Zane, Roberto Dragone

**Affiliations:** Consiglio Nazionale delle Ricerche—Istituto per lo Studio dei Materiali Nanostrutturati c/o Dipartimento di Chimica, ‘Sapienza’ Università di Roma, P. le Aldo Moro 5, 00185 Roma, Italyroberto.dragone@cnr.it (R.D.)

**Keywords:** applied microbiology, white biotechnology, green chemistry, nanostructured materials, diatom nanotechnology, sensoristic devices, drug delivery, theranostics

## Abstract

Nanomaterials are increasingly being used in new products and devices with a great impact on different fields from sensoristics to biomedicine. Biosynthesis of nanomaterials by microorganisms is recently attracting interest as a new, exciting approach towards the development of ‘greener’ nanomanufacturing compared to traditional chemical and physical approaches. This review provides an insight about microbial biosynthesis of nanomaterials by bacteria, yeast, molds, and microalgae for the manufacturing of sensoristic devices and therapeutic/diagnostic applications. The last ten-year literature was selected, focusing on scientific works where aspects like biosynthesis features, characterization, and applications have been described. The knowledge, challenges, and potentiality of microbial-mediated biosynthesis was also described. Bacteria and microalgae are the main microorganism used for nanobiosynthesis, principally for biomedical applications. Some bacteria and microalgae have showed the ability to synthetize unique nanostructures: bacterial nanocellulose, exopolysaccharides, bacterial nanowires, and biomineralized nanoscale materials (magnetosomes, frustules, and coccoliths). Yeasts and molds are characterized by extracellular synthesis, advantageous for possible reuse of cell cultures and reduced purification processes of nanomaterials. The intrinsic variability of the microbiological systems requires a greater protocols standardization to obtain nanomaterials with increasingly uniform and reproducible chemical-physical characteristics. A deeper knowledge about biosynthetic pathways and the opportunities from genetic engineering are stimulating the research towards a breakthrough development of microbial-based nanosynthesis for the future scaling-up and possible industrial exploitation of these promising ‘nanofactories’.

## 1. Introduction

**Figure 1:**
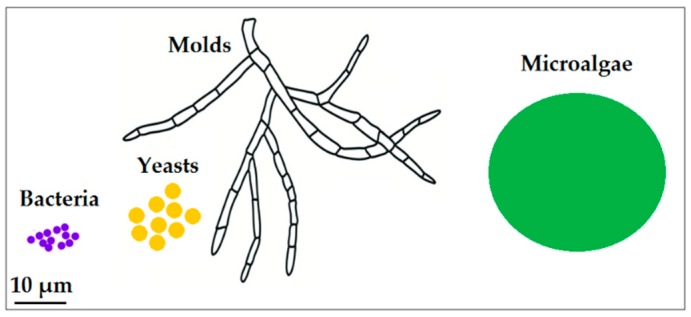
Schematic comparing average sizes of the microorganisms described in this review.

During the period of 2016–2022 the global nanomaterials market is expected to grow with a compound annual growth rate of about 20% or more [1]. One of the major challenges for the global advancement of nanomaterials market is the environmental sustainability of nanomanufacturing processes. Indeed, traditional top-down or bottom-up chemical and physical nanomanufacturing approaches have a greater energy-intensity compared to manufacturing processes of bulk materials. Further, they are often characterized by low process yields (using acidic/basic chemicals and organic solvents), generation of greenhouse gases, and they require specific facilities, operative conditions (e.g., from moderate to high vacuum), and high purity levels of starting materials [2,3,4]. The principles of green chemistry (“the invention, design and application of chemical products and processes to reduce or to eliminate the use and generation of hazardous substances”) combined with white biotechnology (“biotechnology that uses living cells—yeasts, molds, bacteria, plants, and enzymes to synthesize products at industrial scale”) can really contribute to the development of more sustainable industrial processes [5], also for nanomanufacturing. The microbial-mediated biosynthesis of nanomaterials is a promising biotechnological-based nanomanufacturing process that represents a ‘green’ alternative approach to physical and chemical strategies of nanosynthesis [6,7]. The microbial-mediated biosynthesis of metallic (also as alloys), non-metallic, or metal oxides nanoparticles have been reported for many microbial strains of bacteria, yeast, molds, and microalgae [8] (Figure 1).

In addition, some microorganisms have shown the capability to biosynthesize unique nanostructured materials, i.e., biomineralized nanostructures like silicified frustules [9], calcified coccoliths [10], magnetosomes [11], and organic nanomaterials like bacterial nanocellulose [12] exopolysaccharide nanoparticles [13] and bacterial nanowires [14]. The microbial-mediated biosynthesis of nanomaterials has been extensively explored showing different advantages and features including the following: (i) synthetized nanomaterials have defined chemical composition, size and morphology, (ii) biosynthesis is performed at mild physico-chemical conditions, (iii) easily handling and cultivation of microbial cells and possibility of cell culture scale-up, (iv) possibility of in vivo tuning nanomaterial characteristics by changing key parameters of cell culture operational set up or through genetically engineering tools [15]. In order to enable a broad applicability of microbial-mediated biosynthesis of nanomaterials as a real alternative to ‘traditional’ synthetic approaches to nanomanufacturing, many hurdles still need to be overcome: a reduction of polidispersity of nanoparticles, a more complete characterization of biocapping layer agents, effectiveness of removal procedures of biocapping layer and nanomaterials purifications, standardization of microbial cell culture protocols for reproducibility of nanosynthesis processess, as well as production costs and yields. Overeaching the challenge for the development of reliable eco-friendly nanotechnologies for nanomaterial synthesis is of utmost importance for future exploitations of broad-impact nanostructured-based technologies and applications, like innovative optical and electrochemical (bio) sensoristic devices [16] and therapeutic and diagnostic applications of nanostructured materials e.g., for drug delivery, in vivo/in vitro imaging and development of antimicrobial and antitumoral drugs [17,18]. In the first part of this review, we reported an overview of scientific literature (mainly from the last ten years) about in vivo microbial biosynthesis of nanomaterials that have been used for (bio) sensoristic and biomedical purposes. We focused on works that have covered several key aspects of nanomaterials: (i) type of biosynthesis (in some cases post-biosynthesis functionalization), (ii) biosynthetic pathways (presumptive or demonstrated), (iii) characterization, (iv) applications. In the second part, main acquired knowledge, challenges, and potentiality of microbial-mediated biosynthesis has been described.

## 2. Microbial-Mediated Biosynthesis of Nanomaterials for Sensoristic and Biomedical Applications

### 2.1. Bacteria

In the last ten years, bacteria have been used to synthesize inorganic nanomaterials (mainly selenium, gold, and silver nanoparticles) with interesting properties for the development of voltammetric sensoristic devices [19], and third-generation biosensors [20], for possible diagnostic applications [21] like cell imaging and biolabeling [22] and for applications where no surface coat is required, like annealing and thin film formation [23] (Table 1). Bacterial-biosynthesized nanoparticles have mainly shown in vitro antimicrobial activity against some pathogenic bacterial strains [24,25,26,27,28] and properties i.e., antioxidant [29], anti-proliferative, anti-migration [30], anticoagulant [31], and anticancer [26,27,28,29,30,31,32,33]. Biochemical mechanisms which mediate the bacterial biosynthesis of nanoparticles have been proposed or they are currently under investigation. Many of these biochemical mechanisms have been described as part of microbial resistance mechanisms for cellular detoxification which involves changes in solubility of inorganic ions by enzymatic reduction and/or precipitation of soluble toxic to insoluble non-toxic nanostructures. Both extracellular and intracellular biocatalytic synthesis (with and possible excretion) mainly involves oxidoreductase enzymes (e.g., NADH-dependent nitrate reductase, NADPH-dependent sulphite reductase flavoprotein subunit α, and cysteine desulfhydrase) and cellular transporters. Physicochemical processes like biosorption, complexation, nucleation, growth, and stabilization mediated by biomolecules (e.g., proteins and carbohydrates) have also been described. In addition to inorganic nanomaterials, some bacteria genera have shown the ability to biosynthesize very peculiar organic nanostructures. Bacterial nanocellulose is a 3-D network of cellulose nanofibrils produced by aerobic acetic bacteria like those belonging to the genus *Gluconacetobacter*, the most efficient bacteria for nanocellulose biosynthesis. Compared to the nanocrystalline cellulose and nanofibrillated cellulose, bacterial nanocellulose shows higher purity, crystallinity and mechanical stability [34]. Therefore, bacterial nanocellulose is a nanomaterial which has attracted great attention for use in biomedical applications (e.g., as antimicrobial agent, for drug delivery systems and scaffolds for tissue engineering) and on biosensoristic platforms (as nanocomposite and as support for the immobilization of biological recognition elements) [35,36,37]. Exopolysaccharides are microbial extracellular biopolymers with different roles in adhesion of bacterial biofilms and as protection agents. In a recent work a novel self-assembled and spherical nanosized non-glucan exopolysaccharide has been described for bacteria *Lactobacillus plantarum*-605. Results have showed its reducing actions for rapid (30 min.) biosynthesis of good monodispersed gold and silver nanoparticles without any pretreatment or modification [38]. Bacterial nanowires are conductive proteinaceous pilus-like nanostructures involved in extracellular electron transport processes of anaerobic dissimilatory metal-reducing bacteria like *Geobacter* and *Shewanella* genera [39], aerobic bacteria like *Pseudomonas aeruginosa* [40] and aerobic photosynthetic cyanobacteria like *Microcystis* and *Synechocystis* genera [41]. Metallic-like conductivity (due to aromatic amino acids-richness in PilA proteic fibers) and a redox-based conductivity (mediated by cytochrome OmcS present on fibers surface) have been hypothesized for bacterial nanowires in *G. sulfurreducens* [39]. Studies on nanowires *Shewanella oneidensis* MR-1 strain have showed a p-type, tunable electronic behavior with electrical conductivities comparable to moderately doped inorganic semiconductors used in synthetic organic semiconductor-based devices like field-effect transistors [42]. The bacterium *S. oneidensis* have been also described for biosynthesis of gold and silver nanomaterials [23,24]. Bacterial nanowires are also very promising nanostructures in the bioelectronic field for the development of new biomaterial for microbial fuel cells and electrochemical (bio) sensoristic devices i.e., as direct electron transfer mediator between bacteria biofilm and the solid-state electrode surfaces. Different silicon-based electrodes for rapid biochemical oxygen demand (BOD) determination and water integral toxicity monitoring have been described in recent literature [43,44,45]. Bacterial magnetosomes are organic-coated intracellular nanocrystals of Fe_3_O_4_ and/or Fe_3_S_4_, biosynthesized by both magnetotactic and non-magnetotactic bacteria. The composition of magnetic inorganic part is species-specific, and the external organic coating layer is derived from bacterial phospholipid bilayer membrane. The putative functions of protein component of the external organic coating layer in the magnetosome biomineralization process have been hypothesized [11]. Bacterial Fe_3_O_4_ magnetosomes are stable single-magnetic domains permanently magnetic at ambient temperature, possessing peculiar characteristics of high chemical purity, a narrow size range and consistent crystal morphology [46]. Some recent applications include molecular imaging [47], cancer therapy [48], and the development of a chip-based whole-cell biosensor for toxicity assessment [49].

### 2.2. Yeasts and Molds

The research focused on biosynthesis of nanomaterials by fungi, like yeasts and molds, have brought to the coinage of the term ‘myconanotechnology’, in order to refer to a newly emerging domain of nanotechnology. Yeasts are unicellular fungi mainly known in nanosynthesis for their ability to produce semiconductor nanoparticles [8]. Biosynthesis of high water-soluble and biocompatible cadmium telluride quantum dots by model organism yeast *Saccharomyces cerevisiae* have been reported in literature. These cadmium telluride quantum dots have showed interesting characteristics of size-tunable (changing culture time and temperature) emission and photoluminescence quantum yield as good candidate for bio-imaging and bio-labelling applications [50]. *S. cerevisiae* have been also used for biosynthesis of Au–Ag alloy nanoparticles for electrochemical sensors fabrication [51,52], aimed to the determination of paracetamol in tablet samples and vanillin in vanilla bean and vanilla tea sample, respectively (see Table 2). Possible biosynthesis mechanisms of nanoparticles by *S. cerevisiae* could involve membrane bound and cytosolic oxidoreductases as well as extracellular 1,3-β-glucan synthase-mediated formation and growth of nanoparticles [50,51,52,53]. Molds are a large group of microscopic filamentous fungi that include many genera like to *Penicillum*, *Aspergillus*, and *Fusarium*. Compared to bacteria, molds possess many distinctive advantages for biosynthesis of nanomaterials: (i) higher metal tolerance, (ii) higher metal binding and uptake capabilities, (iii) easy culturing and fast growing; (iv) higher extracellular nanosynthesis (mediated by extracellular enzyme, reductive proteins, and secondary secreted metabolites). Extracellular biosynthesis of nanomaterials poses advantages in terms of a possible reuse of cell cultures for new biosynthesis (cell lysis not required) and reduced nanoparticle downstream purification processes [54]. Proposed mechanisms behind fungal synthesis of nanoparticles hypothesized a possible involvement of biomolecules secreted in formation and stabilization of nanoparticles [55], secreted reductases [56,57] and possible trapping of metal ions by electrostatic interaction with positively charged groups in enzymes present in cell wall of the mycelia [58]. In the last ten years, several works have described mold-based biosynthesis of nanoparticles (silver, gold, and tellurium) and quantum dots (zinc sulfide, zinc sulfide with gadolinium, and lead sulfide). These nanoparticles have shown both antibacterial activity [54,57,59] and antitumoral activity [55,56,58] beside possible employment in optical detection of heavy metals and arsenic in water [60,61] (Table 2).

### 2.3. Microalgae

Microalgae are unicellular photosynthetic microorganisms that have attracted significant interest in the field of nanomanufacturing [62] (see Table 3). Microalgae like *Tetraselmis kochinensis*, *Scenedesmus* and *Desmodesmus* have been used for the biosynthesis of noble metal nanoparticles with good antimicrobial activity, useful for applications in biomedical tool designing but also in drug delivery, catalysis and electronics [63,64,65]. For these microalgae, mechanisms described for nanoparticles biosynthesis include phenomena of nucleation, control of dimension, and stabilization of nanoparticle structure, mediated by reducing agents [64], enzymes present in the cell wall cytoplasmic membrane [63], biomolecules like polysaccharides, proteins, polyphenols and phenolic compounds [65]. Mechanisms behind biological mineralization (or biomineralization), i.e., the in vivo inorganic minerals formation, have been extensively studied for possible development of new nanomaterials. Diatoms are unicellular microalgae with very peculiar biomineralized silica cell wall called frustules. Diatom frustules possess a highly periodic and hierarchical 3D-porous micro-nanostructure of different morphology (pennate and centric). Hypotheses about their natural functions include mechanical protection, biological protections, filtration, DNA protection from UV and optimization of light harvesting [66,67,68]. Compared to analogous synthetic mesoporous silica materials, e.g., MCM-4, diatom frustules possess different advantages, including higher biocompatibility, reduced toxicity and easily purification. Diatom frustules also exhibit interesting optical and optoelectronic properties [66,69,70,71,72,73,74]. The abundance of silanol groups (Si-OH) make the diatom frustules surface easily functionalizable (also in vivo), thus allowing to fully exploit the potentiality of structural nanopatterning of diatom frustules [75]. Functionalization with antibodies [72,73,74,75,76,77] and gold nanoparticles [78,79] has been described for different sensitive materials in optical or electrochemical immunosensors. Diatom frustules have showed potential application in drug delivery systems [80,81,82]. Diatomaceous earth (or diatomite) is a large available microfossil material from diatom frustules with extensive commercial use in abrasives or filters. Recently, diatomite—gold nanoparticle nanocomposites have been described for on-chip chromatography and surface-enhanced Raman scattering-based sensoristic devices for the detection of cocaine in biological samples [83] and of histamine in salmon and tuna samples [84]. The “diatom nanotechnology” is a rapidly evolving research field which aims to fully exploit the unique properties of diatom frustules and the great potential of silica biomineralization cellular pathway for the development of new functionalized nanomaterials for emerging applications in sensing, photonic and drug delivery [85]. Another characteristic of diatom frustules is the presence of xanthophyll pigment fucoxanthin. Recent studies have highlighted the active role of fucoxanthin as photo-reducing agent of metal ions to stabilize silver nanoparticles. These silver nanoparticles have showed a significant in vitro antimicrobial activity against *Escherichia coli*, *Bacillus stearothermophilus*, and *Streptococcus mutans* [86] and possible application in optical chemosensing of dissolved ammonia in water samples [87]. Compared to diatoms, microalgae coccolithophores have received less attention. Coccolithophores are calcifying nanoplankton that produces CaCO_3_ microparticles (coccoliths), in form of arrays of nanoscaled substructures. Interesting optical properties (e.g., light scattering) of coccoliths have been described but also some drawbacks including low electrical conductivity, dissolution at low pH values and scarcity of surface functional groups. Despite these, coccoliths morphologies have showed a great applicative potential for nanodevices fabrications, especially following appropriate in vivo or in vitro modification and functionalizations [10,78]. In very recent work the fabrication of an electrochemical aptamer-based sandwich-type biosensor for the detection of type 2 diabetes biomarker Vaspin has been described [88].

## 3. Towards a Large-Scale Applicability: Knowledge, Issues, and Potentiality

The in vivo microbial nanobiosynthesis and possible control and tuning of nanomaterial properties represent a concrete opportunity for future development and promising uses in biosensoristics and biomedical fields. Despite all the advantages, microbial nanotechnology still has very limited uses [89]. Bacteria have showed the ability to synthetize nanomaterials either by extracellular or intracellular mechanisms. These mechanisms generally produce opposite advantages and disvantages in terms of metal nanoparticles dispersity and purification. Extracellularly produced nanoparticles are generally more polydispersed (i.e., with a great variability in size) than intracellularly produced nanoparticles. By contrast, in extracellular nanomaterial productions less downstream extraction/purification steps (e.g., ultrasound treatment and detergent uses) are required. Thus, the extracellular mediated synthesis described for yeast and molds can greatly simplify the purification steps, besides being an advantage for a possible reuse of microorganisms for more biosynthesis cycles. However, the characterization and identification of the enzymes responsible for nanobiosynthesis in molds is still uncomplete. The photoautotrophic metabolism of microalgae and cyanobacteria is based on carbon dioxide (as carbon source), light (as energy source), inorganic nutrients and water. This condition generally reduce the costs of culture media (compared to culture media used for the growth of bacteria, yeasts, and molds) and it can strongly spur the future scaling-up from the laboratory to the industrial scale, also through the design and the development of solar photobioreactors for the fixation (and reduction) of atmospheric carbon dioxide.

### 3.1. Nanoparticles Dispersion and Capping Layers

One of the main challenges in microbial nanobiosynthesis is the control of dispersity of nanostructure materials, which heavily influence electronic and optical properties, and the isolation and purification of plural form. Dispersity, i.e., the size distribution of the nanoparticle population, is a key property that strongly influences the particle’s behavior in fluids. Improvement and optimization of extraction and purification protocols are required, both for intracellular and extracellular biosynthesis: methods like freeze-thawing, osmotic shock and centrifugation could lead to changes in nanoparticle structures as well as aggregation and precipitation phenomena. Through the adoption of suitable strategies, microbial biosynthesis of nanoparticles could be improved. Selection of appropriate microbial strains (in terms of growth rate and biocatalytic activities), optimization of culturing conditions and uses of genetic engineering tools could help to overcome drawbacks linked to slower producing rate and polidispersity (compared to chemical-based nanomanufacturing) [90]. Microbial biosynthetic nanoparticles are characterized by the presence of a capping layer of biomolecules adsorbed on the surface that act as stabilizing agent and biological active layer of nanoparticles [21]. A deep knowledge of capping characteristics, a clear identification of capping agents (mainly peptides like glutathione, metallothioneins, membrane associated proteins etc.), and possible purification of nanoparticles [23] are fundamental for future in vivo medical applications [15,91].

### 3.2. Cell Culture Conditions

For future large-scale productions, costs of culture media for microbial growth should be seriously considered to not limit the applications of microbial biosynthetic nanomaterials. One current example is bacterial nanocellulose whose applications are still limited to a few biomedical devices, mainly because of costs of culture medium [92]. In addition, optimization and standardization of microbial cell culture growth protocols and modifications culture conditions are pivotal for control, tune, and to improve characteristics of microbial biosynthesized nanomaterials. The influence of physico-chemical parameters of cell culture operational set up on nanomaterials biosynthesis have been previously highlighted. These factors include (i) microbial cell concentration; (ii) precursor concentration; (iii) pH; and (iv) temperature. The optimum conditions of pH, temperature, and NaCl concentration have been studied to achieve high purity and high synthesis rate of cadmium selenide nanoparticles by bacterium *Pseudomonas aeruginosa* strain RB. Interestingly, the results of this work have showed that optimum conditions for nanoparticles synthesis did not match with optimum growth conditions for *Pseudomonas aeruginosa* strain RB [93]. Effects of precursor concentration, temperature, and pH on silver nanoparticle synthesis and particle sizes have been described for the bacterium *E. coli* strain DH5α [94]. Other recent examples include: (i) the temperature-dependence of size and monodispersity of silver nanoparticles biosynthesis by mold *Trichoderma viride* [95], (ii) the influence on the type of gold nanostructures synthetized (nanoparticles or nanoplates) and them relative size in yeast *Yarrowia lipolytica* strain NCIM 3589 by changing the proportion of cell concentration and precursor gold salt concentration, (iii) the effect of temperature on gold nanostructures release from cell wall into the aqueous phase [96] and control of bacterial growth kinetics of the bacterium *Morganella psychrotolerans* to achieve shape anisotropy of silver nanoparticles [97]. Elsoud et al. (2018) observed an improvement in tellurium nanoparticles production by a 1 kGy of gamma irradiation of mold *Aspergillus welwitschiae* KY766958 broth culture (compared to the non-irradiated broth culture control). These results have been ascribed to the activation of enzyme (s) involved in biosynthetic pathway [59]. Although the controlling of biomineralization process still remains a challenge, the optimization of frustules morphological properties (e.g., pore sizes and pore density) has been explored by changing operational parameters of experimental setup (e.g., pH, salinity, temperature, nutrient concentration, precursor Si(OH)_4_ concentration, and light regime) [98]. Interestingly Townley et al. (2007) reported alteration in pore sizes of *Coscinodiscus wailesii* frustules when exposed to sublethal concentration of nickel [99]. The possibility of in vivo chemical modification of frustules or other biomineralized structures has been recently described. These in vivo chemical modifications lead to the inorganic elements/compounds-doping of biomineralized structures through the addition to the culture medium of given precursors at sublethal concentrations. Several works described the doping of diatom *Pinnularia* sp. frustules or diatoms *Thalassiosira weissflogii* frustules with titania (TiO_2_) [100,101] nanobiosynthesis containing Si-Ge oxides nanocomb in diatom *Nitzschia* and *Pinnularia* sp. by adding Ge(OH)_4_ or GeO_2_ in the diatom culture medium at photobioreactor scale-productions [102,103,104,105]. Compared to diatoms, possible nanotechnological applications of the calcareous-based shell of marine protozoa foraminifera have not been so widely explored. A recent work described the in vivo preparation of a bionic material through the inclusion of fluorescent magnetite nanoparticles within calcite skeletal structure of the unicellular organism foraminifer *Amphistrigina lesson*. Such in vitro synthetic approach exploited the natural biomineralization process of growth in the presence of magnetic nanoparticles functionalized with a hydroxylated dextran shell [106].

### 3.3. Biochemistry, Molecular Biology, and Genetics

A deeper knowledge of molecular biology and genetic aspects behind microbial nanobiosynthetic pathways is strongly required. For instance, the characterization of not fully understood biochemical mechanisms and a complete identification of extracellular enzymes secreted by filamentous fungi could lead to an improved control in chemical compositions, shapes, and sizes of nanoparticles [55]. The availability of microorganism genome sequences could considerably increase the range of possibilities in genetic manipulation of microorganism to implement the nanoparticles biosynthesis and the in vivo tuning of nanoparticle characteristics. A recent example has come from the study of Zhang et al. (2017), which showed how CdSe quantum dots biosynthesis can be improved through genetic modification of the ATP metabolism pathway in yeast *S. cerevisiae* [107]. Biotechnological approaches based on genetic engineering and recombinant technologies could allow the identification of sequences of gene involved in nanoparticle synthesis and a possible heterologous expression (i.e., controlled expression of one or more gene sequences in a host organism) to enhance nanomaterial production efficiency [91]. The bacterium *E. coli* is a highly efficient model host microorganism that has been exploited as heterologous expression system for phytochelatin synthase and/or metallothionein for the in vivo synthesis of various metal nanoparticles (e.g., CdSeZn, PrGd, CdCs, and FeCo) never synthesized before by chemical methods [108,109] or cadmium selenide quantum dots [110]. Thanks to recent advances in the characterization of biochemical mechanisms involved in bacterial nanocellulose biosynthesis, the future development of new genetically engineered bacterial nanocellulose-producing strains could be achieved. This could eventually lead to a reduction of production costs, an improvement of production yield, and biosynthesis of nanocellulose with new properties, suitable for broader range of technological applications [92,111]. Concerning genetic manipulation of nanostructure-producing microorganisms, Tan et al. (2016) have reported a 2000-fold increase in electrical conductivity and diameter of nanowires filaments produced by model microorganism *Geobacter sulfurreducens*. In this case, genetic modification has been concerned the modification of aminoacidic composition of the carboxyl end of PilA protein, the structural component of bacterial nanowires [112]. The biomineralization process behind magnetosome biogenesis in bacterial species (*Magnetospirillum* species are the most studied) is very complex and not completely elucidated. The mechanism of magnetosome formation has been shown to be under tight genetic control and induced by growth conditions. To date, six different models have been proposed to elucidate magnetosome formation, but they are still not completed [11]. Delalat et al. (2015) have reported a genetically engineered diatom *Thalassiosira pseudonana* (whose genome has been completely sequenced) to incorporate immunoglubulin G-binding domain of protein G (GB1) into the frustules surface. Such antibody-labelled genetically modified diatom enables an in vitro selectively cell targeting and selectively killing of neuroblastoma and B-lymphoma cells [113]. Furthermore, the role of gene Silicanin-1 in the control of biosilica morphology has been recently highlighted in *Thalassiosira pseudonana*, opening new possibilities for future genetic engineering of frustules architectures [114]. A deeper knowledge of biosilicification process as well as the role of organic components of diatom frustules (i.e., proteins silaffins and long-chain polyamines) in biogenesis and formation of nanopatterns in diatom frustules are still a challenge. To date, biocalcification system of coccolithophores remains unclear, even though a very recent genetic and proteomic study about expression of transcripts and proteins in coccolithophore *Emiliania huxleyi* will help future identification and more detailed characterization of molecular mechanisms and metabolic pathways underlying calcification in coccolithophores [115].

## 4. Conclusions

In the light of recent literature herein reported, microbial nanotechnology is a fascinating and booming field for future breakthrough nanomaterial synthesis. Through a ‘green’ and sustainable approach, microbial nanotechnology can really spur innovation in nanomanufacturing with a potential strong impact in several fields, including sensoristics and biomedicine.

## Figures and Tables

**Table 1:** Nanomaterials synthesized by bacteria.

Microorganism	Culture Conditions (Synthesis Time)	Nanomaterial	Characterization	Biosynthetic Pathway	Application	Ref.
*Bacillus subtilis*	Enrichment medium, 35 °C, stirred at 170 rpm + 4 mM Na_2_SeO_3_ (48 h)	Se NPs	50–400 nm; spherical regular morphology; 100 nm uniform single-crystalline; nanowires	Reduction mechanism of SeO_3_^2−^ ions to Se^0^ is yet to be elucidated	H_2_O_2_ sensoristic device	[19]
*Streptomyces minutisclero-ticus* M10A62	5 g of wet bacterial biomass from 120 h cell culture + 1 mM Na_2_SeO_3_, stirred at 200 rpm (72 h)	Se NPs	10–250 nm; spherical shape; crystalline; ζ-potential −19.1 mV	Extracellular synthesis not described	Anti-biofilm, antioxidant activity, antiviral activity against Dengue virus; anti-proliferative activity against HeLa and HepG2 cell lines	[21]
*Pantoea agglomerans* strain UC-32	1% (*v*/*v*) of an overnight cell culture in tryptic soy broth + 1 mM Na_2_SeO_3_, 25 °C (24 h)	Se NPs	<100 nm; spherical shape; amorphous form size vary with culture time (10–24 h);	Intracellular reduction of Se (IV) to Se (0) and subsequent excretion	High antioxidant activity (when stabilized with L-cysteine)	[29]
*Streptomyces bikiniensis* strain Ess_amA-1	1 mL fresh bacteria inoculums (OD_600_ = 0.5 a.u.) in international *Streptomyces* Project 2 medium + 1 mM SeO_2_, 30 °C, stirred at 150 rpm (48 h)	Se NPs	600 nm length, 17 nm diameter	Possible involvement of proteins/enzymes in SeO_2_ reduction nucleation, growth, stabilization of nanorods	In vitro anticancer activity against human breast adenocarcinoma cell line and human liver carcinoma cell line	[32]
*Escherichia coli* DH5α	10 h culture, resuspended in sterile distilled water + 1 mM HAuCl_4_, room temperature (120 h)	Au NPs	25 ± 8 nm; spherical shape; crystalline form (face centered cubic phase)	Extracellular synthesis possibly modulated by sugars or enzymes present onto bacteria surface	Direct electro-chemistry of hemoglobin	[20]
*Shewanella oneidensis* MR-1	Washed cell pellet from a 24 h cell culture + 1 mM HAuCl_4_, 30 °C, stirred at 200 rpm (48 h)	Au NPs	12 ± 5 nm; spherical shape, capping proteins easily removable but not identified	Extracellular synthesis possible electron shuttle-based enzymatic reduction of ionic Au^3+^ to Au^0^	No antibacterial properties/annealing and thin film formation	[23]
*Nocardiopsis* sp. MBRC-48	Cell-free supernatant (from a 96 h cell culture) + 0.9 mM HAuCl_4_, incubated in the dark, 35 °C, stirred at 180 rpm (48 h)	Au NPs	11.57 ± 1.24 nm; spherical shape; face centered cubic; polydispersed without significant structure	Extracellular synthesis using the cell free supernatant, proteins, enzymes and metabolites	High antimicrobial activity against *Staphylococcus aureus* and *Candida albicans*, antioxidant activity and cytotoxic activities	[25]
*Brevibacterium casei*	1 g of wet bacterial biomass + 1 × 10^−3^ M AgNO_3_ + 1 × 10^−3^ M HAuCl_4_, 37 °C, stirred at 200 rpm (24 h)	Au and Ag NPs	Ag 10–50 nm, Au, 0–50 nm; spherical shape, crystalline form (face centered cubic phase)	Intracellular synthesis, possible roles of NADH-dependent nitrate reductase (for Ag NPs) and α-NADPH-dependent sulfite reductase (for Au NPs)	Anti-coagulant properties	[31]
*Shewanella oneidensis* MR-1	∼3–5 g of wet bacterial biomass from 24 h cell culture + 1 mM AgNO_3_, 30 °C stirred at 200 rpm (48 h	Ag NPs	∼2–11 nm spherical shape; crystalline form; ζ-potential = −16.5 mV	Extracellular synthesis by secreted factors (e.g., NADH-dependent reductases, quinines, soluble electron-shuttles)	Antibacterial activity against *Escherichia coli* and *Bacillus subtilis*	[24]
*Lyngbya majuscula* (CUH/Al/MW-150)	100 mg of fresh weight biomass + 9 mM Ag(I) solution (pH 4) incubated in the dark, room temperature (72 h)	Ag NPs	∼5–50 nm; spherical shape, crystalline form (face-centered cubic), smooth surface morphology, both (sonication) ζ-potential = −35.2 mV	Extracellular and intracellular synthesis not described	Effective antibacterial activity against *Pseudomonas aeruginosa*; appreciable anti-proliferative effect on leukemic cells, especially on the REH cell line	[26]
*Streptomyces s.* Al-Dhabi-87	Broth-free cell pellets (14-days cell culture) in sterile distilled water for 1 h; cell removed from the suspension + 1–5 mM AgNO_3_, 37 °C (48 h)	Ag NPs	20–50 nm; spherical shape	Extracellular synthesis possibly via hydrophilic and hydrophobic small metabolites attached on the bacteria cell wall	In vitro antimicrobial activity against *Bacillus subtilis*, *Enterococcus faecalis*, *Staphylo-coccus epi-dermidis*, and multidrug resistant *Staphylococcus aureus* strain	[27]
*Bacillus licheniformis*	2 g of wet bacterial biomass + 1 mM AgNO_3_, 37 °C, stirred at 200 rpm (24 h)	Ag NPs	40 nm to 50 nm	N/A	Possible application as anti-proliferative and anti-migration agent e.g., against diabetic retinopathy, neoplasia and rheumatoid arthritis	[30]
*Escherichia coli* K12 (ATCC 29181)	Bacterial culture (OD_600_ = 0.6 a.u.), Luria Bertani medium + 3 mM CdCl_2_ + 6 mM Na_3_C_6_H_5_O_7_ + 0.8 mM Na_2_TeO_3_, 8 mM C_4_H_6_O_4_S + 26 mM NaBH_4_, 37 °C, stirred at 200 rpm (24 h)	CdTe QDs	∼2–3 nm; uniform size, cubic crystals; strong fluorescence emission shift with increasing quantum dots size, capping proteins were not identified but enhance QDs biocompatibility; ζ-potential = −19.1 mV	Extracellular synthesis possibly via protein-assisted nucleation biosynthesis	Possible application in vitro cell imaging (demonstrated on HeLa cells) and bio-labeling	[22]
*Acetobacter xylinus GIM1.327*	Static culture in polysaccharides enriched medium, 30 °C (120 h)	Bacterial nanocellulose nanofibrils impregnated with Ag-NPs	Nanoporous three-dimensional network structure with a random arrangement of ribbon-shaped microfibrils without any preferential orientation; 2 to 100 nm (Ag NPs)	Intracellular-extracellular synthesis via enzymes glucokinase, phosphoglucomutase, UDPG, pyro-phospho-rylase and cellulose synthase	In vitro pH-responsive antimicrobial activity against *Escherichia coli* ATCC 25922, *Staphylococcus aureus* ATCC 6538, *Bacillus subtilis* ATCC 9372 and *Candida albicans* CMCC(F) 98001	[28,35]
*Acetobacter xylinum*	N/A	Ag NPs and bacterial nano-paper composite	AgNPs 10–50 nm	Intracellular-extracellular synthesis of bacterial nanocellulose via enzymes glucokinase, phosphoglucomutase, UDPG, pyro-phospho-rylase and cellulose synthase AgNPs synthesis via direct chemical reduction of Ag^+^ mediated by baring hydroxyl groups of bacterial nanocellulose	Optical detection of cyanide ion and 2-mercaptobenzo-thiazole in water samples	[35,36]
*Acetobacter xylinum*	Static culture containing 50 g/L glucose, 5 g/L yeast extract, 5 g/L (NH_4_)_2_SO_4_, 4 g/L KH_2_PO_4_ and 0.1 g/L MgSO_4_·7H_2_O, 28 °C (366 h)	Nanocompositesof bacterial nanocellulose with AgNP, Au-NPs CdSe@ZnS quantum dots functionalized with biotinylated antibodies, aminosilica-coated lanthanide-doped up-conversion NPs	(bacterial nanocellulose) 45 ± 10 nm (fiber mean diameter); estimated length > 10 μm	Intracellular-extracellular synthesis via enzymes glucokinase, phosphoglucomutase, UDPG, pyro-phospho-rylase and cellulose synthase	Optical detection of methimazole, thiourea, cyanide, and iodide and *Escherichia coli*; possible uses in analytes pre-concentration platform	[35,37]
*Bacillus marisflavi* GS3	200 mg biomass + 2.4 × 10^−5^ M graphene oxide dispersion mixture, 37 °C (72 h)	Reduced graphene oxide nanosheets	~4.3 nm (average thickness), significant reduction of GO (assessed by XRD analysis); several layers stacked on top of one another like silky sheets of paper (SEM image)	Extracellular synthesis not described	Inhibition of cell viability, reactive oxygen species (ROS) generation, and membrane integrity alteration in MCF-7 cell line	[33]
*Magnetospirillum magneticum AMB-1* (Genetically modified)	Anaerobically grown in 5 ml/L of Wolfe’s mineral solution (without iron), + 5 mM KH_2_PO_4_ + 10 mM NaNO_3_ + 0.85 mM C_2_H_3_NaO_2_ + 0.2 mM C_6_H_8_O_6_ + 2.5 mM C_4_H_6_O_6_ + 0.6 mM Na_2_S_2_O_3_, pH 6.9; cell pellets were resuspended in 20 mM HEPES + 1 mM EDTA + 8% glycerol + 0.9% NaCl, pH 7.5	Magnetosome (bio-mineralized iron-oxide nanoparticles coated by a biological membrane)	Magnetosome membrane modified with Venus-RGD protein as specific and sensitive molecular imaging probe	Natural mechanism of magneto-somes formation (biomineralization) + genetic modification for Venus protein- RGD peptide expression	Contrast agent for in vivo magnetic resonance-based molecular imaging	[47]
*Magnetospirillum magneticum* strain *AMB-1*	Micro-anaerobically grown in a similar culture medium of [47]	Whole inactive magnetotactic bacteria γ-Fe_2_O_3_ magnetosomes chains individual γ-Fe_2_O_3_ magnetosomes	Magnetosomes chains (length) ∼150 or ∼300 nm; individual magnetosomes mean size ∼45 nm; well-crystallized monodomain with a ferromagnetic behavior at physiological temperature	Natural mechanism of magneto-somes formation + genetic modification for Venus protein- RGD peptide expression	Antitumoral activity against MDA-MB-231 breast cancer cells under alternating magnetic field stimulation	[48]
*Magnetospirillum gryphiswaldense* strain *MSR-1*	Micro-anaerobically grown in a similar culture medium of [47] and [48] + 50μM Fe(III) citrate	Chains of magnetosomes	Magnetosome membrane modified with Red-emitting Click Beetle luciferase (CBR)	Natural mechanism of magneto-somes formation + genetic modification for red-emitting click beetle luciferase expression	Toxicity assay on microfluidic chip for the detection of toxicity effect on membrane by DMSO and TCDCA	[49]

**Table 2:** Nanomaterials synthesized by yeasts and molds.

Microorganism	Culture Conditions (Synthesis Time)	Nanomaterial	Characteristics (Average Size, Morphology, etc.)	Biosynthetic Pathway	Application	Ref.
*Saccharomyces cerevisiae*	Aerobic two days growth in a modified Czapek’s medium, 5 °C; aliquot of cell suspension (OD_600_ = 0.6) + 3 mM CdCl_2_ + 0.8 mM Na_2_TeO_3_ + 1.5 mM CH_3_SO_3_H + 2.6 mM NaBH_4_, stirred at 500 rpm (N/A)	CdTe QDs	2.0–3.6 nm; cubic zinc blende crystals	Extracellular synthesis not described	Good candidate for bio-imaging and bio-labelling applications	[50]
*Aspergillus welwitschiae* KY766958	Growth in Czapek’s medium; pH 7.3 ± 0.2, 30 °C for 7 days shaken at 150 rpm + 2 mmol K_2_TeO_3_ (48 h)	Te NPs	60.80 nm; oval to spherical shape	Mechanism not described	Antibacterial activity against *E. coli* and methicillin resistant *Staphylococcus aureus* (MRSA)	[59]
Commercially available instant dry yeast (Angel Yeast Co.—Yichang, China)	Sucrose solution (5 g/L) + instant dry yeast (600 mg), 30 °C for 24 h; cells pellet in sterile water (10^6^ cells/mL) + AgNO_3_ solution + HAuCl_4_ solution (final concentrations N/A), 30 °C. (24 h)	Au–Ag alloy NPs	Reduced metallic form (XPS analysis); large superficial area and desirable conductivity (electrochemical impedance spectroscopy)	Extracellular synthesis not described	Electrochemical sensor for paracetamol	[51]
		Au–Ag alloy NPs	9–25 nm	Extracellular synthesis not described	Electrochemical sensor for vanillin	[52]
*Humicola* sp.	MGYP medium, pH 9, shaken at 200 rpm, 50 °C; harvested mycelial mass + 1 mM AgNO_3_, shaken at 200 rpm, 50 °C (96 h)	Ag NPs	5–25 nm; spherical shape; face centered cubic crystals	Extracellular synthesis through a possible involvement of biomolecules secreted by the fungus	In vitro cytotoxicity against NIH3T3 mouse embryonic fibroblast cell line and MDA-MB-231 human breast carcinoma cell line	[55]
*Fusarium oxysporum* f. sp. *lycopersici*	5 days growth, potato dextrose broth, 28 °C; filtered biomass + 1 mM AgNO_3_, 28 °C, dark condition (120 h)	Ag NPs	5 to 13 nm; spherical shape; face centered cubic crystals	Extracellular synthesis, possible involvement of a secreted reductase	Antibacterial activity against pathogenic bacteria *Escherichia coli* and *Staphylococcus aureus*; antitumoral activity against human breast carcinoma cell line MCF-7	[56]
*Penicillium brevicompactum* KCCM 60390	72 h growth, potato dextrose broth, 30 °C, shaken at 200 rpm; filtered biomass (5 g) in Milli-Q sterile deionized water and agitated, 72 h at 200 rpm, 30 °C; supernatant from filtered biomass + 1 mM HAuCl_4_, shaken at 200 rpm, dark condition 30 °C (N/A)	Au NPs	(live cell filtrate) 25–60 nm; spherical shape; 20–80 nm (potato dextrose broth), spherical and triangular and hexagonal shape (culture supernatant broth) 20 to 50 nm; well dispersed and uniform in shape and size; good stability against aggregation after 3 months	Extracellular synthesis; possible ion trapping on the fungal cells surface via electrostatic interaction; possible involvement of organic reagents used for the microbial cultivations as potential reducing agents	Inhibitory effect and cytotoxicity against mouse cancer C_2_C_12_ cell lines	[58]
*Trichoderma harzianum* (SKCGW008)	72 h cultured spores in wheat bran broth media, 28 °C shaken at 180 rpm; supernatant + 0.5% (*w/v*) of low molecular weight chitosan in agitation (30 min)	Chitosan NPs	90.8 nm; spherical shape; amorphous structure	Extracellular synthesis via enzyme secreted (not identified)	Antioxidant activity; bactericidal activity against *Staphylococcus aureus* and *Salmonella enterica*; biocompatibility (no cytotoxic effect on murine fibroblast NIH-_3_T_3_ cells)	[57]
*Aspergillus flavus*	Growth in potato dextrose broth, 28 °C, 115 rpm; harvested fungal biomass + 3 mM ZnSO_4_, 27 °C, 200 rpm; for ZnS:Gd nanoparticle 0.3 M Gd(NO_3_)_3_ (96 h)	ZnS and ZnS: Gd NPs	Nanocrystalline and a narrow size distribution: 12–24 nm spherical (ZnS): for and 10–18 nm (ZnS:Gd)	Extracellular synthesis not described	Optical detection of Pb (II), Cd (II), Hg (II), Cu (II), and Ni (II) in water	[60]
*Aspergillus flavus*	Growth in potato dextrose broth + 0.5 mM Pb(CH_3_COO)_2_ + 6.4 mM Na_2_S, 30 °C, 115 rpm (120 h)	PbS NPs	35–100 nm; cubic crystal	Extracellular synthesis not described	Optical detection of As (III) in water	[61]

**Table 3:** Nanomaterials synthesized by microalgae.

Microorganism	Culture Conditions (Synthesis Time)	Nanomaterial	Characteristics (Average Size, Morphology, Modification)	Biosynthetic Pathway	Application	Ref.
*Tetraselmis kochinensis*	Guillard’s Marine Enrichment medium at 28 °C, 200 rpm, 15 days, light condition. 10 g of washed harvested cells + 1 mM HAuCl_4_, 200 rpm, 28–29 °C (48 h)	Au NPs	5–35 nm; spherical and triangular shape	Intracellular synthesis; possible reduction via enzymes present in the cell wall and in the cytoplasmic membrane	Various applications including catalysis, electronics and coatings	[63]
*Scenedesmus* sp. (IMMTCC-25)	Growth in Modified Bold Basal medium, 28 ± 2 °C, 16:8 h light: dark cycle,126 rpm; washed pelleted biomass (harvested in the logarithmic growth phase) + 5 mM AgNO_3_, 28 °C in the same growth conditions (72 h)	Ag NPs	(living cells) 3–35 nm; spherical shape, highly crystalline cluster; (raw algal extract) (5–10 nm), spherical shape; (boiled algal extract) >50 nm; less stable; colloidal stability >3 months (assessed UV-Vis measures at 420 nm)	Intracellular synthesis not described. Extracellular synthesis (raw algal extracts); reducing and stabilizing agents involved in nucleation points and size control	Good antimicrobial activity against *Streptococcus mutans* and *Escherichia coli* (boiled cell extract)	[64]
*Desmodesmus* sp. *(KR 261937)*	Growth in BG-11 medium for 15–20 days, 12:12 h light: dark cycle, 28 ± 2 °C, 120 rpm; centrifuged harvested biomass + 5 mM AgNO_3_, 28 °C in the same growth condition (72 h)	Ag NPs	(whole cells); 10–30 nm; ζ-potential = −20.2 mV; (raw algal extract) 4–8 nm; ζ-potential = −19.9 mV; (boiled algal extract) 3–6 nm; ζ-potential = −14.2 mV	Intracellular synthesis not described Extracellular synthesis: biocomponents (e.g., polysaccharides, proteins, polyphenols and phenolic compounds) possibly involved in control of dimension and stabilization	Antibacterial effect against *Salmonella* sp. and *Listeria monocytogenes*; antifungal activity against *Candida parapsilosis*	[65]
*Coscinodiscus concinnus Wm.*	One-week growth (cell density 10^6^ cells mL^−1^) in silicate-enriched seawater media, 18–20 °C, 12:12 h light: dark cycle	Biogenic silica (frustules) modified with murine monoclonal antibody UN1	Green photoluminescence (peaked between 520 and 560 nm) of silanized frustules	Natural silicification process (bio-mineralization)	Using the biogenic silica photo-luminescence for immunosensors development	[72]
*Cyclotella* sp.	Growth in Harrison’s Artificial Seawater Medium enriched with f/2 nutrients + 0.7 mM Na_2_SiO_3_, 22 °C 14:10 h light: dark cycle. The cell suspension was subcultured at 10% *v*/*v* every 14 days (336 h)	Biogenic silica (frustules) functionalized with IgG	~200-nm (perimetrical pores) ~100-nm (linear arrays of pores from the center to the rim) at the base of each ~100-nm pore, a thin layer of silica containing four to five nanopores of ~20-nm diameter	Natural silicification process (bio-mineralization)	Label-free photoluminescence-based immunosensor	[73]
*Coscinodiscus wailesii*	Growth in F/2 seawater medium, 20 °C, continuous photoperiod	Functionalized biogenic silica (frustules)	100–200 μm	Natural silicification process (bio-mineralization)	Electrochemical immunosensor for the detection of C-reactive protein and myelo-peroxidase in buffer and human serum samples	[75]
*Cosinodiscus argus* and *Nitzschia soratensis*	Growth in F/2 medium, 20 °C, 12:12 h light: dark cycle. The culture media volume was doubled every week to keep high the diatom reproduction rate About 4000 cells/ml and about 5.5 × 10^5^ cell/ml for *C. argus* and *N. soratensis* respectively); (about 1000 h)	Multi-layered package array of biogenic silica (frustules) functionalized with purified primary rabbit IgG	*C. argus* 80–100 μm uniformly distributed sub-micron elliptical holes (~170–300 nm) and nanopores (~90–100 nm); *N. soratensis* ~10–15 μm (long axis) and ~5μm (short axis) with nanopores (60–80 nm)	Natural silicification process (bio-mineralization)	Optical immunochip for fluorophore-labeled donkey anti-rabbit IgG detection	[76]
*Pseudostaurosira trainorii*	Growth in F/2 medium + silica 7 mg mL^−1^, under aeration 12:12 h light: dark cycle	Biogenic silica (frustules) integrated with Au NPs functionalized with 5,5′-dithiobis (2-nitrobenzoic acid) + anti-interleukin-8 antibodies	4–5 μm; 98% silica Perpendicular oriented rows of 4–5 pores (100–200 nm) decreasing in size towards the central axis; neighboring rows separated by ~450 nm; neighboring pores in a row separated by ~100 nm	Natural silicification process (bio-mineralization)	Surface-enhanced Raman scattering immunosensor for the detection of interleukin 8 in blood plasma	[77]
*Pinnularia* sp. (UTEX #B679)	Growth in Harrison’s artificial seawater medium + 0.5 mM Na_2_SiO_3_, 22 °C, 14:10 h light: dark cycle for 21 days. (336 h)	Biogenic silica (frustules) functionalized with anti- 2,4,6-TNT single chain variable fragment derived from the monoclonal antibody 2G5B5	Ellipsoidal shape with major axe ~20 μm minor axe ~6 μm; pores in rectangular array (~200 nm diameter) spaced 300–400 nm apart. 4–5 nanopores (~50 nm diameter) at the base of each pore	Natural silicification process (bio-mineralization)	Label-free photo-luminescence quenching -based sensor for 2,4,6-trinitro-toluene detection	[77]
*Aulacoseria* sp.	N/A	Biogenic silica (frustules) coated with gold (multiple layers of Au particles)	5–10 μm cylindrical-shaped frustules	Natural silicification process (bio-mineralization)	Functional support for surface-enhanced Raman scattering sensor	[78]
*Melosira preicelanica*	N/A	biogenic silica (frustules) tailored with Au NPs	~20 nm cylindrical-shaped frustules	Natural silicification process (bio-mineralization)	Detection of bovine serum albumin and mineral oil by surface-enhanced Raman spectroscopy	[79]
*Coscinodiscus concinnus*	Same conditions reported in [70]	Biogenic silica (frustules) loaded with streptomycin	Homogeneous size distribution with a radius of 220 ± 15 µm	Natural silicification process (biomineralization	Drug delivery	[80]
*Thalassiosira weissflogii* CCAP strain 1085/10	Growth in silicate-enriched seawater media, 18–20 °C, 12:12 h light: dark cycle, final cell density 10^6^ cells mL^−1^ (168 h)	Biogenic silica (frustules)	Mainly composed of separated valves, porosity and hierarchically ordered nanostructure; luminescent and nanostructured silica shells, combining the dye photoluminescence with the photonic silica nanostructure	Natural silicification process (bio-mineralization)	Loading and delivery of fluoro-quinolone ciprofloxacin	[81]
Fossil diatoms	N/A	Biogenic silica (frustules) integrated with 50–60 nm gold nanoparticles	~400 μm (width of the diatomite channels porous); disk-shaped; extremely high confinement of the analyte and increase the concentration of target molecules at the sensor surface; photonic crystals (substrate for surface-enhanced Raman scattering) with 50–60 nm Au NPs	N/A	On-chip chromatography-surface-enhanced Raman scattering -based microfluidic label-free device for cocaine detection in biological samples	[84]
Fossil diatoms	N/A	Biogenic silica (frustules) integrated with 50–60 nm Au nanoparticles	10 to 30 μm; dish-shaped with two-dimensional periodic pores; thickness of the diatomite layer on the glass ~20 μm, (one-third of that of a commercial Thin Layer Chromatography, chip) photonic crystals (substrate for surface-enhanced Raman scattering	N/A	On-chip chromatography-surface-enhanced Raman scattering -based microfluidic label-free device for histamine in salmon and tuna	[85]
*Amphora*-46	Growth in F/2 medium made with filter sterile brackish water (salinity 3%, pH 8.2), 30 °C, 16:8 h light: dark cycle, 130 rpm; Aqueous cell extract + 2 mM AgNO_3_, 35–40 °C (30 h)	polycrystalline Ag NPs	20–25 nm	Extracellular synthesis; photosynthetic pigment fucoxanthin acts as a reducing agent	Antimicrobial activity against *Escherichia coli*, *Bacillus stearothermophilus,* and *Streptococcus mutans*	[86]
*Emiliania huxleyi* strain CCMP371	Growth in Artificial seawater (ASW) + f/2 nutrients (without added Si), 20 °C, 12:12 h light: dark cycle, 130 rpm. Cells were harvested at late exponential phase	Aptamer-modified coccolith electrodeposited on the screen-printed Au electrode	N/A	Natural calcification process (coccolitho-genesis)	Aptamer-based sandwich-type electrochemical biosensor for Vaspin (type 2 diabetes biomarker)	[88]
